# Measles Outbreak among Previously Immunized Adult Healthcare Workers, China, 2015

**DOI:** 10.1155/2016/1742530

**Published:** 2016-04-20

**Authors:** Zhengyi Zhang, Yuan Zhao, Lili Yang, Changhong Lu, Ying Meng, Xiaoli Guan, Hongjin An, Meizhong Zhang, Wenqin Guo, Bo Shang, Jing Yu

**Affiliations:** ^1^Department of General Medicine, Lanzhou University Second Hospital, Gansu 730030, China; ^2^Department of Public Health, Lanzhou University Second Hospital, Gansu 730030, China; ^3^Department of Cardiology, Lanzhou University Second Hospital, Gansu 730030, China

## Abstract

Measles is caused by measles virus belonging to genus* Morbillivirus* of the family Paramyxoviridae. Vaccination has played a critical role in controlling measles infection worldwide. However, in the recent years, outbreaks of measles infection still occur in many developing countries. Here, we report an outbreak of measles among healthcare workers and among the 60 measles infected patients 50 were healthcare workers including doctors, nurses, staff, and medics. Fifty-one patients (85%) tested positive for IgM antibodies against the measles virus and 50 patients (83.3%) tested positive for measles virus RNA. Surprisingly, 73.3% of the infected individuals had been previously immunized against measles. Since there is no infection division in our hospital, the fever clinics are located in the Emergency Division. In addition, the fever and rash were not recognized as measles symptoms at the beginning of the outbreak. These factors result in delay in isolation and early confirmation of the suspected patients and eventually a measles outbreak in the hospital. Our report highlights the importance of following a two-dose measles vaccine program in people including the healthcare workers. In addition, vigilant attention should be paid to medical staff with clinical fever and rash symptoms to avoid a possible nosocomial transmission of measles infection.

## 1. Introduction

Measles is a highly infectious respiratory disease that occurs worldwide, which mainly causes morbidity and mortality among children. Its causative agent, the measles virus, is a single stranded negative sense RNA virus belonging to the Paramyxoviridae family [1]. The clinical symptoms of measles include high fever, a maculopapular rash, conjunctivitis, cough, and coryza; furthermore, its complications can include pneumonia, blindness, and brain damage [2]. The incubation period of the disease ranges from 6 to 21 days, and its transmission modes include airborne droplet transmission and direct contact with the respiratory secretions of an infected individual or their fomites [2]. An infected individual can disseminate the virus to others 2 days before and 5 days after the onset of their own clinical symptoms. Vaccine immunization has played an important role in preventing, controlling, and eradicating infectious diseases such as poliomyelitis. Since the 1960s, a single dose of liquid measles vaccine has been administered to infants aged >8 months in China [3]. Starting in 1978, a national Expanded Program on Immunization (EPI) was initiated to ensure that all infants received one dose of measles vaccine at the age of 8 months. In 1985, an amended two-dose vaccination schedule was established, in which the first dose of vaccine is administered at the age of 8 months and the second dose at the age of 7 years [4]. In 2005, China took action to eliminate measles in the Western Pacific Region by 2012 (a goal set by the World Health Organization) and changed the age for administration of the second vaccine dose from 7 years to 18 months [5]. Use of the measles vaccine has resulted in substantial reductions in the incidence and mortality of measles worldwide, including China [6]. In addition to the EPI, a nationwide measles Supplementary Immunization Activity (SIA) was initiated in 2010 to close immunization gaps among children and in other populations [3]. In 2012, the SIA program had helped to reduce the incidence of measles to its lowest recorded level (6183 cases, 4.6 cases/million total population) [7]. However, in recent years, there have been several reports of measles infections in adults, raising concerns about the current immunization programs [8, 9]. Here, we report for the first time a measles outbreak among healthcare workers in mainland China.

## 2. Case Presentation

On March 16, 2015, a patient admitted to the Emergency Department with high fever was diagnosed as having a measles infection. On the day of March 20, 2016, three nurses from Emergency Department who had direct and (or) indirect contact with the patient displayed similar clinical signs including fever and rash that were determined to be positive for measles infection. The hospital noticed that it might be a break of measles infection with possibility of nosocomial transmission, and the first case on March 16, 2015, was suspected to be the source of this outbreak. Immediately, a series of measures for the prevention and control of nosocomial measles according to Guidelines of the People's Republic of China on the Prevention and Control of Infectious Diseases have been set up as follows.

*Measures for the Prevention and Control of Nosocomial Measles*. Consider the following:

1. Setting up isolation wards in a well-ventilated old building.
2. Enhancing ventilation, isolation, and disinfection measures in isolation areas.
3. Isolating those patients with great transmission potentials including severe-ill patients and students.
4. Providing measles immunization to a total of 2,400 hospital staff.
5. Close monitoring of the measles infected patients, paying much more attention to patients with severe complications, and canceling multidisciplinary team meetings and treatment.

**Figure 1:**
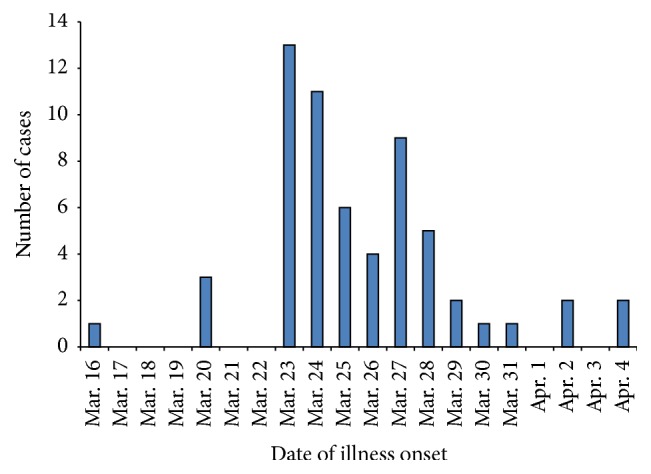
An epidemic curve shows the number of cases during the measles outbreak in Lanzhou University Second Hospital between March 16, 2016, and April 4, 2015. The *x*-axis represents date of illness onset; the *y*-axis represents the number of cases.

First, the isolation ward was set up with a well-ventilated system to prevent possible transmission among community people and the first patient (case number 1) was hospitalized two weeks before discharge. Second, Emergency Department is the unit with serious and multiple illness in which the healthcare staff from other departments who went to consultation directly or indirectly were exposed to the Emergency Department patients with measles; they were infected with measles and/or spread it to other staff in their departments. Therefore, more medical staffs from other departments with similar symptoms and close contact with the known measles cases were searched. Third, reimmunization probably played important roles in containing measles transmission and a total of 2400 hospital staff were given a measles immunization before March 27, 2015. Since then, new measles case numbers began to decrease and the last two cases were reported on April 4, 2016 (Figure 1). Among 20 patients admitted in isolation wards, only 6 patients were treated. Due to the mild symptoms in most hospital staff patients, they chose to stay home and no new case was reported among those persons contacted with patients who stayed home. The study was approved by Institutional Review Board (IRB) of the Lanzhou University Second Hospital and written patient consent was obtained under Review Board approval number 2015A-077. Verbal consent was obtained from all participants using an information sheet approved by the IRB of the Lanzhou University Second Hospital.

Patients with clinical symptoms including fever, rash, and/or conjunctivitis were defined as suspected measles cases. Between March 16 and April 4, 2015, both serum and throat swab samples were gathered from 102 suspected measles cases and then tested for measles virus IgM using IgM ELISA test kit (Zhuhai SEZ Haitai Biological Pharmaceuticals Co., Ltd., Zhuhai, China) and for measles virus RNA using a real-time reverse transcription PCR kit (Jiangsu BioPerfectus Technologies Co., Ltd., Jiangsu, China). Finally, 60 suspected measles cases were confirmed as the diagnosis of measles infection among whom there were 51 (85.00%) cases positive for measles IgM, 50 (83.33%) for measles virus RNA, and 41 (68.33%) for both measles IgM and virus RNA. Among the 60 positive cases, 39 (65.00%) and 21 (35.00%) were in female and male patients, respectively. Seven cases (11.66%) were aged 1–18 years, 50 cases (83.33%) were aged 21–40 years, and 3 cases (5.01%) were aged >40 years (Table 1). There were 31 (51.66%) nurses, 11 (18.33%) hospital staff members, 7 (11.66%) doctors, 1 (1.69%) medic, and the remaining 10 (16.66%) hospital patients (Table 1). Thus 83.33% of measles cases were healthcare workers. The majority of cases displayed one or more of the following symptoms: Koplik spots in 36 patients (60%), catarrh in 51 patients (85%), and conjunctivitis in 50 patients (83.3%). Only a few patients had severe symptoms, which included pneumonia in 2 patients (3.3%) and liver dysfunction in 6 patients (10%) (Tables 1 and 2).

## 3. Discussion

The results of a recent study of measles infections in China indicated that measles usually occurred in young unvaccinated children [7]; however, the measles outbreak in our hospital mainly involved adults aged between 20 and 40 years. Surprisingly, 44 of the 60 infected patients (73.33%) had received a single dose measles vaccination during their childhood, and only one patient (1.67%) received a second immunization at the age of 18 years. Thus while >70% of the patients had been previously immunized against measles, they remained susceptible to infection with the measles virus. This finding suggests that the level of protection provided by a single measles immunization may drop to baseline and cease to protect against the disease. The possibility of one-dose primary vaccine failure in the patients that led to their susceptibility to measles can not be excluded. Moreover, two previous studies reported that measles outbreaks in Beijing and Hangzhou mainly occurred among people aged >15 years. However, 87.6% and 66.7% of the adults infected in those two outbreaks, respectively, had unknown vaccination histories [8, 9]. More importantly, several measures should be immediately taken to prevent and control measles infection and transmission in hospitals. These measures include increasing the room ventilation in all departments, promptly isolating suspected cases from other patients, establishing a program for evaluating the measles immune status of all hospital healthcare workers, and providing a second vaccination when appropriate and all measles-susceptible individuals should be vaccinated immediately following contact with a suspected case of measles [10].

## 4. Conclusion

In summary, our study reports a measles outbreak that occurred among adult healthcare workers who were previously underimmunized against measles. It highlights that healthcare workers need to be fully vaccinated through a 2-dose measles vaccine program, which could greatly prevent nosocomial outbreak of measles. Equally important, fever clinics should be separated from other divisions in the hospital. Vigilant attention should be paid to the patients with the clinical fever and rash symptoms and early isolation and definite diagnosis of the measles suspected medical staff patients to avoid a possible nosocomial transmission of measles infection.

## Figures and Tables

**Table 1:** Summary of cases diagnosed with measles in the outbreak.

*Gender*	
Female (*n*, %)	39 (65.00%)
Male (*n*, %)	21 (35.00%)
*Age*	
1–18 years (*n*, %)	7 (11.66%)
19–40 years (*n*, %)	50 (83.33%)
>40 years (*n*, %)	3 (5.01%)
*Prevalent location of the measles*	
New building of the hospital (*n*, %)	60 (100%)
Old building of the hospital (*n*, %)	0 (0.00%)
*Occupation*	
Nurse (*n*, %)	31 (51.66%)
Staff (*n*, %)	11 (18.33%)
Clinical patient (*n*, %)	10 (16.66%)
Doctor (*n*, %)	7 (11.66%)
Medic (*n*, %)	1 (1.69%)
*Vaccination history*	
Vaccination (*n*, %)	44 (73.33%)
Unknown (*n*, %)	12 (20.00%)
No vaccination (*n*, %)	4 (6.67%)
*Positive rate of the test method*	
MV IgM (*n*, %)	51 (85.00%)
MV PCR (*n*, %)	50 (83.33%)
Both (*n*, %)	41 (68.33%)
*Clinical manifestation*	
Koplik spots (*n*, %)	36 (60.00%)
Catarrh (*n*, %)	51 (85.00%)
Conjunctivitis (*n*, %)	50 (83.33%)
Complication (*n*, %)	
Pneumonia (*n*, %)	2 (3.33%)
Laryngitis (*n*, %)	1 (1.67%)
Liver dysfunction (*n*, %)	6 (10.00%)

**Table 2:** General information and clinical manifestations of measles patients.

Case number	Date of onset	Gender	Age (years)	Occupation	Vaccination history	MV IgM	MV PCR	Clinical manifestation					
								Koplik spots	Catarrh	Conjunctivitis	Pneumonia	Laryngitis	Liver dysfunction
1	Mar. 16	Female	33	ED patient	No	Positive	Positive	Yes	Yes	Yes	Yes	No	Yes
2	Mar. 20	Female	29	ED nurse	Yes	Positive	Positive	Yes	Yes	Yes	No	No	Yes
3	Mar. 20	Female	27	ED nurse	No	Positive	Positive	Yes	Yes	Yes	No	No	Yes
4	Mar. 20	Male	31	ED nurse	Yes	Positive	Positive	Yes	No	Yes	No	No	No
5	Mar. 23	Male	26	Logistical staff	Yes	Positive	Positive	No	Yes	Yes	No	No	No
6	Mar. 23	Female	25	Surgical nurse	Yes	Positive	Positive	No	Yes	No	No	No	No
7	Mar. 23	Male	36	Logistical staff	Unknown	Positive	Positive	No	Yes	Yes	No	No	No
8	Mar. 23	Female	31	Surgical nurse	Unknown	Positive	Positive	Yes	No	Yes	No	No	No
9	Mar. 23	Female	41	Logistical staff	No	Positive	Positive	No	Yes	Yes	No	No	No
10	Mar. 23	Male	32	Neurological doctor	Unknown	Positive	Positive	No	Yes	Yes	No	No	No
11	Mar. 23	Female	26	Hematological nurse	Yes	Positive	Positive	Yes	Yes	No	No	No	No
12	Mar. 23	Male	32	Logistical staff	Yes	Positive	Positive	No	Yes	Yes	No	No	No
13	Mar. 23	Male	27	Logistical staff	Yes	Positive	Positive	No	Yes	Yes	No	No	No
14	Mar. 23	Male	25	Pediatric nurse	Yes	Positive	Positive	No	No	No	No	No	No
15	Mar. 23	Male	29	Logistical staff	Yes	Positive	Positive	No	Yes	Yes	No	No	No
16	Mar. 23	Female	27	Respiratory nurse	Yes	Positive	Positive	Yes	Yes	Yes	No	No	No
17	Mar. 23	Female	24	Ophthalmological nurse	Yes	Positive	Positive	Yes	Yes	Yes	No	No	No
18	Mar. 24	Male	27	Logistical staff	Yes	Positive	Positive	No	Yes	Yes	No	No	No
19	Mar. 24	Male	24	Cardiology nurse	Yes	Positive	Negative	Yes	Yes	Yes	No	No	No
20	Mar. 24	Female	23	Clinic nurse	Yes	Positive	Negative	Yes	Yes	Yes	No	No	No
21	Mar. 24	Female	24	Clinic nurse	Yes	Positive	Negative	Yes	Yes	Yes	No	No	No
22	Mar. 24	Female	25	Clinic nurse	Yes	Positive	Negative	Yes	No	Yes	No	No	No
23	Mar. 24	Male	30	Neurological doctor	Yes	Positive	Negative	No	Yes	Yes	No	No	No
24	Mar. 24	Female	22	Surgical nurse	Yes	Positive	Negative	No	Yes	No	No	No	No
25	Mar. 24	Female	25	Surgical nurse	Yes	Positive	Negative	Yes	Yes	Yes	No	No	No
26	Mar. 24	Female	24	Neurological nurse	Yes	Positive	Negative	Yes	Yes	Yes	No	No	No
27	Mar. 24	Female	25	Surgical nurse	Yes	Positive	Negative	No	Yes	Yes	No	No	No
28	Mar. 24	Male	1	Hematological patient	Yes	Positive	Positive	Yes	Yes	Yes	Yes	Yes	Yes
29	Mar. 25	Female	27	Obstetrical patient	Yes	Positive	Positive	Yes	Yes	Yes	No	No	Yes
30	Mar. 25	Female	30	ICU nurse	Unknown	Positive	Positive	No	No	No	No	No	No
31	Mar. 25	Female	26	Neurosurgical nurse	Yes	Positive	Positive	No	Yes	Yes	No	No	No
32	Mar. 25	Female	28	Urological nurse	Unknown	Positive	Positive	Yes	Yes	Yes	No	No	No
33	Mar. 25	Female	19	VIP medical nurse	Yes	Positive	Positive	Yes	Yes	Yes	No	No	No
34	Mar. 25	Female	32	Cardiology nurse	Unknown	Positive	Positive	No	Yes	No	No	No	No
35	Mar. 26	Female	13	Pediatric patient	Yes	Positive	Positive	Yes	Yes	Yes	No	No	No
36	Mar. 26	Male	30	Chinese medicine doctor	Unknown	Positive	Positive	Yes	Yes	Yes	No	No	Yes
37	Mar. 26	Female	27	VIP medical nurse	Yes	Positive	Positive	Yes	No	Yes	No	No	No
38	Mar. 26	Female	26	Cardiology nurse	Yes	Positive	Negative	Yes	Yes	Yes	No	No	No
39	Mar. 27	Male	16	ED patient	Yes	Positive	Positive	Yes	Yes	Yes	No	No	No
40	Mar. 27	Male	30	Urological doctor	Unknown	Positive	Positive	No	Yes	No	No	No	No
41	Mar. 27	Female	29	Logistical staff	Yes	Negative	Positive	Yes	Yes	Yes	No	No	No
42	Mar. 27	Male	21	Medical student	Yes	Negative	Positive	No	Yes	Yes	No	No	No
43	Mar. 27	Male	14	ED patient	Yes	Positive	Positive	Yes	Yes	No	No	No	No
44	Mar. 27	Male	25	Logistical staff	Yes	Positive	Positive	Yes	Yes	Yes	No	No	No
45	Mar. 27	Female	27	Cardiology nurse	Yes	Positive	Positive	Yes	Yes	Yes	No	No	No
46	Mar. 27	Female	38	ED patient	Yes	Positive	Positive	No	Yes	Yes	No	No	No
47	Mar. 27	Male	1	Pediatric patient	Yes	Positive	Positive	Yes	Yes	Yes	No	No	No
48	Mar. 28	Female	27	Clinic nurse	Yes	Positive	Positive	No	Yes	Yes	No	No	No
49	Mar. 28	Female	16	ED patient	Yes	Positive	Positive	Yes	No	No	No	No	No
50	Mar. 28	Female	22	Neurological nurse	Yes	Positive	Positive	Yes	Yes	Yes	No	No	No
51	Mar. 28	Male	13	Pediatric patient	Yes	Positive	Positive	Yes	Yes	No	No	No	No
52	Mar. 28	Female	29	Hematological nurse	Unknown	Positive	Positive	No	Yes	Yes	No	No	No
53	Mar. 29	Female	43	Logistical staff	No	Positive	Positive	No	Yes	Yes	No	No	No
54	Mar. 29	Female	27	Pediatric nurse	Yes	Negative	Positive	Yes	Yes	Yes	No	No	No
55	Mar. 30	Female	38	Nephrology doctor	Unknown	Negative	Positive	Yes	Yes	Yes	No	No	No
56	Mar. 31	Female	25	Urological nurse	Yes	Negative	Positive	No	No	Yes	No	No	No
57	Apr. 2	Female	47	Logistical staff	Unknown	Negative	Positive	Yes	Yes	Yes	No	No	No
58	Apr. 2	Female	29	Pediatric nurse	Yes	Negative	Positive	No	Yes	Yes	No	No	No
59	Apr. 4	Male	32	Logistical staff	Unknown	Negative	Positive	Yes	Yes	No	No	No	No
60	Apr. 4	Female	23	Neurological nurse	Yes	Negative	Positive	No	No	Yes	No	No	No

ED: Emergency Department; ICU: intensive care unit; VIP: very important person.
